# SARS-CoV-2 Pandemic Impact on Pediatric Emergency Rooms: A Multicenter Study

**DOI:** 10.3390/ijerph17238753

**Published:** 2020-11-25

**Authors:** Luigi Matera, Raffaella Nenna, Valentina Rizzo, Francesca Ardenti Morini, Giuseppe Banderali, Mauro Calvani, Matteo Calvi, Giorgio Cozzi, Elisabetta Fabiani, Raffaele Falsaperla, Ahmad Kantar, Marcello Lanari, Riccardo Lubrano, Beatrice Messini, Antonio Augusto Niccoli, Pietro Scoppi, Vincenzo Tipo, Fabio Midulla, on behalf of the CICERO Group

**Affiliations:** 1 Department of Maternal, Infantile and Urological Sciences, Sapienza University of Rome, 00161 Rome, Italy; luigi.matera91@gmail.com (L.M.); raffaella.nenna@uniroma1.it (R.N.); valentina.rizzo@hotmail.it (V.R.); 2 Pediatric Unit, Sant’Eugenio Hospital, 00144 Rome, Italy; francesca.ardentimorini@aslroma2.it; 3 Department of Clinical Paediatrics, San Paolo Hospital, University of Milan, 20142 Milan, Italy; giuseppe.banderali@unimi.it; 4 Operative Unit of Pediatrics, San Camillo-Forlanini Hospital, 00151 Rome, Italy; mi5660@mclink.it; 5 Pediatric Emergency Department, Papa Giovanni XXIII Hospital, 24127 Bergamo, Italy; mcalvi@asst-pg23.it; 6 Institute for Maternal and Child Health Burlo Garofolo, 34137 Trieste, Italy; giorgio.cozzi@burlo.trieste.it; 7 Department of Pediatric Emergency, Gaspare Salesi Hospital, Azienda Ospedaliera Ospedali Riuniti, 60123 Ancona, Italy; elisabetta.fabiani@ospedaliriuniti.marche.it; 8 General Pediatrics and Pediatric Acute and Emergency Unit, Vittorio Emanuele University Hospital, 95121 Catania, Italy; raffaelefalsaperla@hotmail.com; 9 Pediatric Unit, Istituti Ospedalieri Bergamaschi, Gruppo Ospedaliero San Donato, 24059 Ponte San Pietro, Italy; kantar@centropediatricotosse.com; 10 Pediatric Emergency Unit, Scientific Institute for Research and Healthcare, Sant’Orsola Hospital, 40138 Bologna, Italy; marcello.lanari@unibo.it; 11 Pediatric Unit, Department of Maternal and Child Health, Santa Maria Goretti Hospital, Sapienza University of Rome, 04100 Latina, Italy; riccardo.lubrano@uniroma1.it; 12 Pediatric Unit, San Giovanni Battista Hospital, 06034 Foligno, Italy; bmessini@alice.it; 13 Pediatric Unit, Fabrizio Spaziani Hospital, 03100 Frosinone, Italy; dr.aniccoli@gmail.com; 14 Pediatric Unit, San Matteo degli Infermi Hospital, 06049 Spoleto, Italy; p.scoppi@icloud.com; 15 Pediatric Emergency Department, Santobono-Pausilipon Hospital, 06049 Napoli, Italy; enzotipo@libero.it

**Keywords:** air communicable infections, emergency rooms, lockdown measures, pediatric, pandemic, SARS-CoV-2

## Abstract

From 9 March to 3 May 2020, lockdown was declared in Italy due to the severe acute respiratory syndrome coronavirus 2 (SARS-CoV-2) pandemic. Our aim was to evaluate how the SARS-CoV-2 pandemic and related preventive strategies affected pediatric emergency rooms (ERs) during this period. We performed a retrospective cohort multicenter study, comparing the lockdown period to the corresponding period in 2019. We examined 15 Italian pediatric ERs in terms of visit rates, specific diagnoses (grouped as air communicable diseases and non-air communicable diseases), and triage categories. During the lockdown period, ER admissions decreased by 81% compared to 2019 (52,364 vs. 10,112). All ER specific diagnoses decreased in 2020 and this reduction was significantly higher for air communicable diseases (25,462 vs. 2934, *p* < 0.001). Considering the triage category, red codes remained similar (1% vs. 1%), yellow codes increased (11.2% vs. 22.3%), and green codes decreased (80.3% vs. 69.5%). We can speculate that social distancing and simple hygiene measures drastically reduced the spread of air communicable diseases. The increase in yellow codes may have been related to a delay in primary care and, consequently, in ER admissions.

## 1. Introduction

The first SARS-CoV-2 outbreak was described in Wuhan, China, in December 2019. Since then, SARS-CoV-2 has spread throughout five continents and the WHO declared the condition as a pandemic on 11 March 2020 [1]. On 20 February 2020, the first SARS-CoV-2 cases were reported in Italy in the city of Codogno, located close to Milan. Current available Italian data, updated to 23 November 2020, reported 1,378,394 cases and 48,106 (3.5%) deaths [2]. The first pediatric case of SARS-CoV-2 infection was described in Shenzhen, China, on 20 January 2020 [3]. Until 18 November 2020, 149,219 (12.1%) pediatric cases were reported in Italy, with eight fatalities [2].

SARS-CoV-2 transmission primarily occurs through aerosolized droplets [3,4]. Moreover, a fecal-oral transmission may also occur [5,6,7,8]. Thus, the most frequent way that children are exposed to this condition is through close contact with a sick family or household member [9], even if in-school transmission has also likely contributed to SARS-CoV-2 outbreaks [10,11,12]. SARS-CoV-2 has been also detected in untreated wastewater [13,14,15].

There is a large consensus that children typically present with a less severe clinical picture compared to adults and that they appear to have a more favorable prognosis [16,17,18,19,20,21,22,23,24,25,26,27,28,29,30,31,32,33,34,35,36].

Unfortunately, Italy was one of the most SARS-CoV-2-affected countries in the world, with most of the cases and fatalities registered in the northern part, particularly in two regions: Lombardy and Veneto. In order to face the pandemic, the Italian government took several preventive measures that largely affected children’s lives. On 9 March 2020, the Italian prime minister declared lockdown for the first time in Italian history: factories, schools, pubs, shopping malls, and restaurants were closed; only supermarkets remained open, providing daily necessities; smart working was implemented and leaving home was forbidden except for extreme necessities [37,38]. These measures even affected children: they were not allowed to go to school, see their friends, or practice their hobbies, which led to a great shift in their daily habits.

In this multicenter study, we sought to evaluate how the SARS-CoV-2 pandemic and related preventive strategies, such as social distancing and hygiene measures, affected pediatric emergency rooms (ERs) in Italy during the lockdown period in terms of ER visit rates, specific ER diagnoses, and triage categories. As a secondary aim, we evaluated the differences in the SARS-CoV-2 pandemic impact on ER admissions between high- and low-incidence areas.

## 2. Materials and Methods

**Figure 1:**
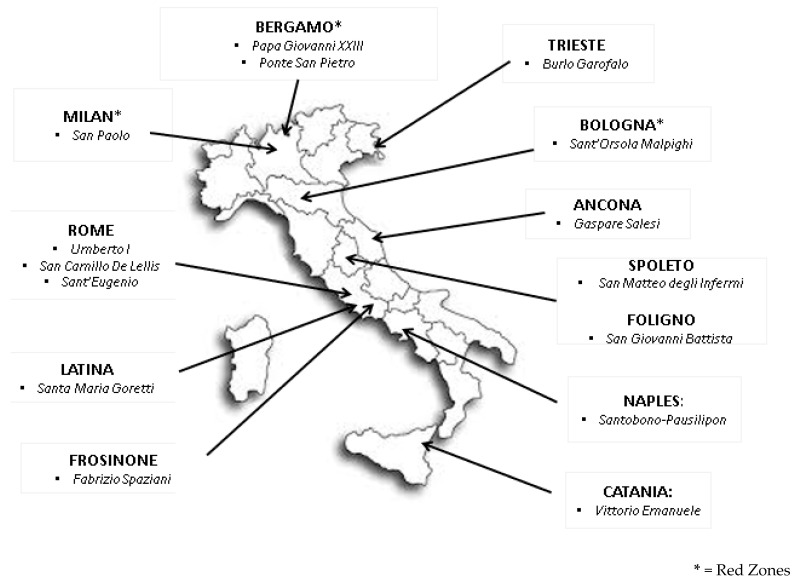
Pediatric emergency rooms (ERs) included in the study.

We performed a retrospective multicenter study involving 15 Italian pediatric ERs, belonging to 12 cities throughout 8 different regions, from northern to southern Italy (Figure 1).

We performed a multistage sampling procedure. In the first stage, 9 of the 20 Italian regions were randomly selected from northern, central, southern, and insular Italian areas. In the second stage, we randomly selected 15 public hospitals from these regions, thus providing a representative sample of the entire Italian population. To quantify the contribution of each hospital in our statistical analysis, we collected the total number of ER admissions in 2019. From January 1 to December 31, 2019, these hospitals registered a total of 311,412 ER admissions, which represented more than 10% of the total pediatric ER admissions that year in Italy.

We considered the so-called lockdown period in Italy [37,38] as 9 March to 3 May 2020, and we compared ER admissions during this period with those during the corresponding timeframe in 2019.

We evaluated 16 ER specific diagnoses, based on the primary discharge diagnoses ruled out in ER according to the ICD9-CM codes. These diagnoses were grouped into two different categories as follows: air communicable and non-air communicable diseases. In the group of air communicable diseases, we included upper and lower respiratory infections, gastroenterological infections, and exanthematous diseases, and we assumed that these conditions were related to airborne transmission. Non-air communicable diseases included accidents, cardiovascular, dermatological diseases, endocrinological disorders, fever, and surgical pathologies, as well as hematological, nephrological, neurological, neuropsychiatric, oncological, ophthalmological, and rheumatological diseases because we assumed that these conditions were not related to airborne transmission. Fever was included in this second category because, if it occurs without any other signs or symptoms, it is more likely related to non-airborne infections, such as urinary tract infections.

We recognized four different triage categories, from the least to the most severe: white, green, yellow, and red [39]. Since the end of 2019, some regions, including Lazio, adopted new triage colors and defined five new triage categories with different priority codes: red (immediate access), orange (access in 15 min), blue (access in 60 min), green (access in 120 min), and white (access in 240 min) [40]. To standardize our data, we combined the orange and blue codes as yellow codes.

We divided our Italian cohort into two different groups. We defined high- and low-incidence areas based on the Italian government’s Decree of the President of the Council of Ministers (Decreto del Presidente del Consiglio dei Ministri) published on 11 March 2020 [37,38] and the Italian National Institute of Health (Istituto Superiore di Sanità (ISS)), which reports Italian SARS-CoV-2-related data daily [2]. This document identified three Italian regions, Emilia-Romagna, Lombardy, and Veneto, as red zones (high-incidence areas) for COVID-19. These were the regions that first experienced the lockdown measures in Italy, including schools’ closure and the ban on people leaving their homes [37,38]: three cities (Bergamo, Milan, and Bologna) were considered high-incidence areas, the red zones; the other nine cities (Rome, Naples, Trieste, Catania, Latina, Frosinone, Spoleto, Foligno, and Ancona) were considered low-incidence areas.

We used IBM SPSS Statistics to analyze our data. We compared the ER admission rate, ER specific diagnoses in terms of air communicable diseases and non-air communicable diseases, and triage categories in 2019 vs. 2020 with chi-square tests.

A *p* value < 0.05 was considered as statistically significant.

## 3. Results

Comparing total ER admissions from 9 March to 3 May 2019, with those from 9 March to 3 May 2020, we observed a reduction rate of 81% (52,364 vs. 10,112 admissions, respectively) (Table 1).

**Figure 2:**
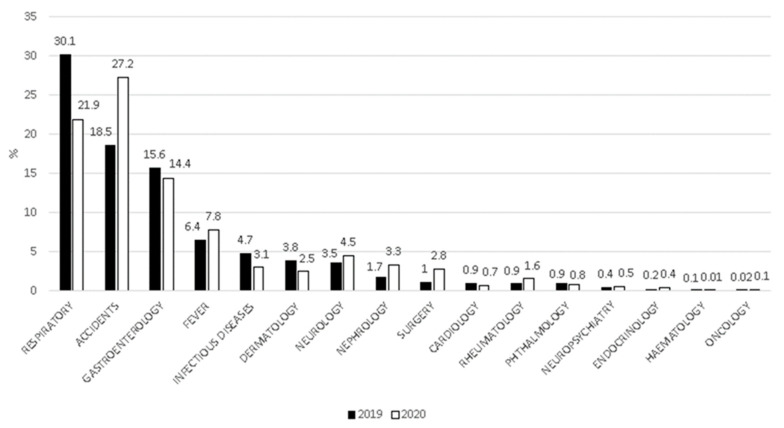
Proportion of pediatric ER diagnoses from 9 March to 3 May 2019, and the same period in 2020.

We encountered a significant reduction in all 16 clinical categories in the 2020 period compared to 2019 (Supplementary Tables). Nevertheless, for some categories, we observed a relative increase in percentage when compared with the total number of admissions per reference periods (Figure 2).

The decrease in air communicable diseases was significantly higher compared to non-air communicable diseases (25,462 vs. 2934 and 26,902 vs. 7178, *p* < 0.001) (Table 2).

**Figure 3:**
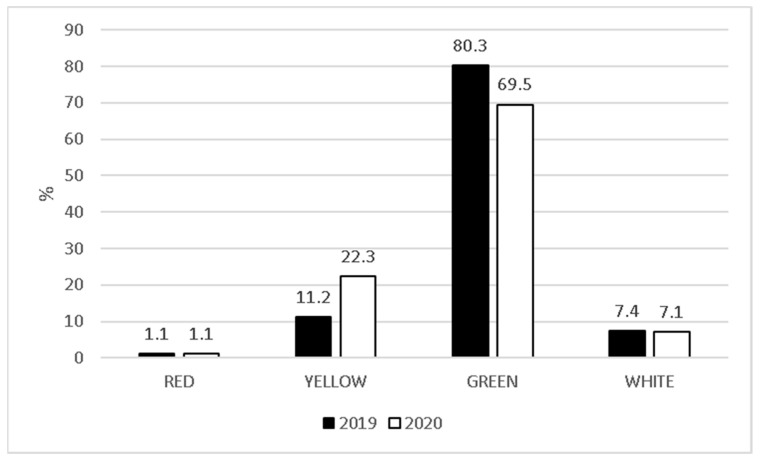
Triage categories from 9 March to 3 May 2019, and the same period in 2020.

ER admissions in each triage category decreased in 2020. Our data showed 408 vs. 97 red codes, 5678 vs. 1580 yellow codes, 40,707 vs. 7339 green codes, and 5533 vs. 1072 white codes in 2019 vs. 2020, respectively. In contrast, considering the proportion of each category over the total number of admissions per reference period, while white codes (7.4% vs. 7.1%) and red codes (1% vs. 1%) remained almost the same, yellow codes increased (11.2% vs. 22.3%), and green codes decreased (80.3% vs. 69.5%) (Figure 3).

Comparing high- and low-incidence areas, in 2020, we found the same reduction as in total ER admissions (−81% and −82%, respectively). Results confirmed the decrease in the absolute numbers of each triage category. According to percentages, no differences were observed in red codes between high- and low-incidence areas. On the contrary, in high-incidence areas compared to low-incidence areas, we demonstrated a less pronounced increase in yellow codes (10.4% vs. 12.6% and 10.9% vs. 16.4%, respectively) and a more marked decrease in green codes (81.0% vs. 73.2% and 76.8% vs. 72.5%, respectively) (*p* < 0.001) (Table 3).

## 4. Discussion

To the best of our knowledge, this is the first multicenter study that aimed to understand how the SARS-CoV-2 pandemic and related preventive strategies affected pediatric ERs in Italy. Evaluating ER admissions in 15 hospitals throughout Italy, we studied a sample potentially representative of the entire Italian population, comparing two reference periods: 9 March to 3 May 2019, and 9 March to 3 May 2020 (the so-called lockdown period) [37,38].

The main result of our study was that total ER admissions in 2020 decreased by 81%. Our results are similar to previous studies that have demonstrated the same trend in both child and adult patients [41,42,43,44,45,46,47,48,49,50,51]. We can speculate that social-distancing measures, the use of face masks, hand washing, and the complete closure of social activities may have contributed not only to the reduction of SARS-CoV-2 diffusion [52,53,54,55,56,57,58,59,60,61,62,63] but also to the reduction of the spread of other diseases, particularly of acute communicable diseases, as confirmed in a recent study [41]. Acute communicable diseases represent the most common clinical conditions in children; in fact, up to 11 respiratory tract infections per year in infancy, 8 episodes per year at pre-school age, and 4 episodes per year at school age could be considered as normal [64]. Our results are also reinforced by several studies that have shown a reduction in the exacerbation of chronic conditions during lockdown period [65], such as asthma [66,67,68,69,70,71,72,73] and cystic fibrosis [74]. The reduction in total ER admissions can be also explained by parents’ fear of leaving their home and of being infected in the hospital [50,75,76], and the explicit order to avoid going to the ER unless in cases of extreme medical necessity [37,38]. Considering the rate of single admissions per reference period, we observed some important differences. The percentage of children with air communicable diseases, such as acute respiratory, gastrointestinal, and infectious diseases, significantly decreased in 2020 [41]. On the other hand, the percentage of children with non-air communicable diseases, such as accidents and neurological, surgical, or urological problems, significantly increased in 2020 [41]. These results should be considered with relative caution, as they may depict an erroneous picture of reality. In fact, the percentage reduction of some diseases determined the percentage increase of others. For this reason, it could seem that the incidence of these diseases has increased. In contrast, our study showed that all of the 16 clinical categories were reduced during the lockdown period.

Every year in Italy, approximately 3 million children are admitted to ER [77]. Approximately 0.5–1% are classified as red codes and 10–12% as yellow codes, meaning that approximately 20,000–30,000 children seek medical advice for life-threatening clinical conditions and about 300,000 children present with serious conditions each year. Nevertheless, approximately 70–80% of ER admissions are categorized as green codes. This considerable number of non-urgent patients with acute clinical conditions, which could be dealt with in an outpatient setting, has necessarily led to the well-known and harmful overcrowding of ER departments [77]. During the lockdown period, each triage category decreased. We may justify this decreasing trend, at least in part, because of the significant reduction in the spread of air communicable diseases [41] and in outdoor accidents, which typically represent the main clinical presentation in pediatric ERs. Moreover, we cannot rule out the fact that the strict preventive measures adopted in Italy [37,38] and parents’ fear of exposing their children to SARS-CoV-2 at the hospital [50,75,76] may have played an important role in this reduction. This raises concerns about what will happen next fall when respiratory viruses will start to circulate again and people will be even more afraid of the possibility of SARS-CoV-2 infection. Interestingly, the percentage of infants with yellow codes increased during the lockdown period, which was probably due to delayed admissions at the ER because of the lack of primary care and parents’ fear of exposing their children to SARS-CoV-2 [78,79,80,81,82]. Delayed admissions at the ER led to severe consequences for the children’s health. In a recent report, the most common delayed ER presentations in the UK and Ireland were diabetes mellitus, sepsis, and malignancies [79]. A total of nine deaths were considered to be causally related to delayed ER admissions in this survey [79]. Thus, in the past lockdown months, children with chronic diseases were at a higher risk of developing severe medical complications because of the inability to access healthcare and medical facilities as usual [78,79,80,81,82]. Another probable consequence that may have negatively impacted patients’ health is inappropriate drug intake due to the lack of easily available medical assistance [75].

Comparing high- and low-incidence areas, we found that total ER admissions showed the same reduction between 2019 and 2020 (−81% and −82%, respectively). No differences were found in red-code percentages. Meanwhile, in high-incidence areas compared to low-incidence areas, yellow codes showed a less pronounced increase (10.4% vs. 12.6% and 10.9% vs. 16.4%, respectively) and, on the contrary, green codes showed a more marked decrease (81.0% vs. 73.2% and 76.8% vs. 72.5%, respectively). We may conclude that fear played a crucial role in this case because the total number of SARS-CoV-2-infected patients was much higher in the red zones. Presumably, the anxiety of being infected by SARS-CoV-2 at home was greater than taking the risk of being infected in the hospital. Thus, children were brought to the ER even in cases of mild but suggestive symptoms of SARS-CoV-2 infection. On the contrary, in low-incidence areas, the fear of being infected in the hospital setting was greater than that of contracting the infection in their own homes, leading to a delay in ER admissions.

This study has some limitations. First, no direct measures of social distancing or other strategies were taken in order to evaluate their contribution for reducing the spread of SARS-CoV-2 and other airborne infections, as other authors have done in previous studies [41,52,63]. Moreover, social distancing measures, the use of face masks, hand washing, and the complete closure of social activities were assumed to be followed. Finally, we did not investigate parents’ feelings about keeping their children at home in spite of medical problems due to the fear of the pandemic. We focused the analysis on the lockdown period when several social distancing measures were implemented simultaneously and we compared data to the same time span in 2019. Thus, in evaluating our data, it is clear that the distancing measures have played a crucial role in limiting the effects of the pandemic.

## 5. Conclusions

The SARS-CoV-2 pandemic and related preventive measures have heavily influenced pediatric ER admissions in Italy. We can speculate that social distancing and simple hygiene measures drastically reduced the circulation of air communicable diseases. It will be interesting to see whether these measures will be effective during the reopening of social activities.

The increase in yellow codes during the lockdown period may be related to a lack of primary care that has consequently resulted in a delay in ER admissions.

## Figures and Tables

**Table 1:** ER admissions reduction between 9 March and 3 May 2019, and the same period in 2020.

Hospitals	2019	2020	Decrease
Umberto I—Rome	3735	674	−82%
San Camillo—Rome	2240	400	−82%
Sant’Eugenio—Rome	1744	90	−95%
Santa Maria Goretti—Latina	1321	220	−83%
Burlo-Garofolo—Trieste	4127	1111	−73%
Vittorio Emanuele—Catania	2000	208	−90%
Gaspare Salesi—Ancona	4044	895	−78%
San Paolo—Milan	2234	189	−92%
Ponte San Pietro—Bergamo	1814	169	−91%
Papa Giovanni XIII—Bergamo	4191	762	−82%
Sant’Orsola Malpighi—Bologna	3893	1083	−72%
Santobono—Naples	16,797	3869	−77%
San Matteo degli Infermi—Spoleto	247	57	−77%
Fabrizio Spaziani—Frosinone	2757	262	−90%
San Giovanni Battista—Foligno	1220	123	−90%
Total	52,364	10,112	−81%

**Table 2:** Air communicable vs. non-air communicable diseases.

Diseases	2019 (*n* = 52,364)	2020 (*n* = 10,112)	*p* Value
Air communicable diseases	25,462 (48.6%)	2934 (29%)	*p* < 0.001
Non-air communicable diseases	26,902 (51.4%)	7178 (71%)	

**Table 3:** Comparison in triage categories between high- and low-incidence areas.

Triage Categories		High-Incidence Areas	Low-Incidence Areas	*p* Value
RED	2019	71 (0.6%)	337 (0.8%)	0.79
	2020	18 (0.8%)	79 (1.0%)	
YELLOW	2019	1267 (10.4%)	4411 (10.9%)	<0.001
	2020	279 (12.6%)	1301 (16.4%)	
GREEN	2019	9824 (81.0%)	30,883 (76.8%)	<0.001
	2020	1613 (73.2%)	5726 (72.5%)	
WHITE	2019	958 (7.9%)	4575 (11.4%)	<0.001
	2020	302 (13.7%)	770 (9.7%)	

## Supplementary Materials

The following are available online at https://www.mdpi.com/1660-4601/17/23/8753/s1: Supplementary Tables.

## References

[1] World Health Organization (2020). WHO Director—General’s Openings Remarks at the Media Briefing on COVID-19. https://www.who.int/director-general/speeches/detail/who-director-general-s-opening-remarks-at-the-media-briefing-on-covid-19---11-march-2020.

[2] Istituto Superiore di Sanità Epicentro. Epidemia COVID-19. https://www.epicentro.iss.it/coronavirus/sars-cov-2-dashboard.

[3] Chan J.F.-W., Yuan S., Kok K.-H., To K.K.-W., Chu H., Yang J., Xing F., Liu J., Yip C.C.-Y., Poon R.W.-S. (2020). A familial cluster of pneumonia associated with the 2019 novel coronavirus indicating person-to-person transmission: A study of a family cluster. Lancet.

[4] Cai J., Wang X., Zhao J., Ge Y., Xu J., Tian H., Chang H., Xia A., Wang J., Zhang J. (2020). Comparison of Clinical and Epidemiological Characteristics of Asymptomatic and Symptomatic SARS-CoV-2 Infection in Children. Virol. Sin..

[5] Xu Y., Li X., Zhu B., Liang H., Fang C., Gong Y., Guo Q., Sun X., Zhao D., Shen J. (2020). Characteristics of pediatric SARS-CoV-2 infection and potential evidence for persistent fecal viral shedding. Nat. Med..

[6] Gu J., Han B., Wang J. (2020). COVID-19: Gastrointestinal Manifestations and Potential Fecal–Oral Transmission. Gastroenterology.

[7] Singh T., Heston S.M., Langel S.N., Blasi M., Hurst J.H., Fouda G.G., Kelly M.S., Permar S.R. (2020). Lessons From COVID-19 in Children: Key Hypotheses to Guide Preventative and Therapeutic Strategies. Clin. Infect. Dis..

[8] Xiao F., Tang M., Zheng X., Liu Y., Li X., Shan H. (2020). Evidence for Gastrointestinal Infection of SARS-CoV-2. Gastroenterology.

[9] Bialek S., Gierke R., Hughes M., McNamara L.A., Pilishvili T., Skoff T. (2020). Coronavirus Disease 2019 in Children—United States, February 12–April 2, 2020. MMWR Morb. Mortal. Wkly. Rep..

[10] Fontanet A., Tondeur L., Madec Y., Grant R., Besombes C., Jolly N., Pellerin S.F., Ungeheuer M.-N., Cailleau I., Kuhmel L. (2020). Cluster of COVID-19 in Northern France: A Retrospective Closed Cohort Study. SSRN Electron. J..

[11] Stein-Zamir C., Abramson N., Shoob H., Libal E., Bitan M., Cardash T., Cayam R., Miskin I. (2020). A large COVID-19 outbreak in a high school 10 days after schools’ reopening, Israel, May 2020. Eurosurveillance.

[12] Torres J.P., Piñera C., De La Maza V., Lagomarcino A.J., Simian D., Torres B., Urquidi C., Valenzuela M.T., O’Ryan M. (2020). Severe Acute Respiratory Syndrome Coronavirus 2 Antibody Prevalence in Blood in a Large School Community Subject to a Coronavirus Disease 2019 Outbreak: A Cross-sectional Study. Clin. Infect. Dis..

[13] Ahmed W., Angel N., Edson J., Bibby K., Bivins A., O’Brien J.W., Choi P.M., Kitajima M., Simpson S.L., Li J. (2020). First confirmed detection of SARS-CoV-2 in untreated wastewater in Australia: A proof of concept for the wastewater surveillance of COVID-19 in the community. Sci. Total. Environ..

[14] La Rosa G., Iaconelli M., Mancini P., Ferraro G.B., Veneri C., Bonadonna L., Lucentini L., Suffredini E. (2020). First detection of SARS-CoV-2 in untreated wastewaters in Italy. Sci. Total. Environ..

[15] Kitajima M., Ahmed W., Bibby K., Carducci A., Gerba C.P., Hamilton K.A., Haramoto E., Rose J.B. (2020). SARS-CoV-2 in wastewater: State of the knowledge and research needs. Sci. Total. Environ..

[16] Cristiani L., Mancino E., Matera L. (2020). Will children reveal their secret? The coronavirus dilemma. Eur. Respir. J..

[17] Cao Q., Chen Y.-C., Chen C.-L., Chiu C.-H. (2020). SARS-CoV-2 infection in children: Transmission dynamics and clinical characteristics. J. Formos. Med. Assoc..

[18] Larsen B., Da S., Al G. (2020). Faculty Opinions recommendation of Potential Maternal and Infant Outcomes from (Wuhan) Coronavirus 2019-nCoV Infecting Pregnant Women: Lessons from SARS, MERS, and Other Human Coronavirus Infections. Fac. Opin. Post Publ. Peer Rev. Biomed. Lit..

[19] Castagnoli R., Votto M., Licari A., Brambilla I., Bruno R., Perlini S., Rovida F., Baldanti F., Marseglia G.L. (2020). Severe Acute Respiratory Syndrome Coronavirus 2 (SARS-CoV-2) Infection in Children and Adolescents. JAMA Pediatr..

[20] Lu X., Zhang L., Du H., Zhang J., Li Y.Y., Qu J., Zhang W., Wang Y., Bao S., Li Y. (2020). SARS-CoV-2 Infection in Children. N. Engl. J. Med..

[21] Cavallo F., Rossi N., Chiarelli F. (2020). Novel coronavirus infection and children. Acta Bio-Med. Atenei Parm..

[22] Götzinger F., Santiago-García B., Noguera-Julián A., Lanaspa M., Lancella L., Carducci F., Gabrovska N., Velizarova S., Prunk P., Osterman V. (2020). COVID-19 in children and adolescents in Europe: A multinational, multicentre cohort study. Lancet Child Adolesc. Health.

[23] Dong Y., Mo X., Hu Y., Qi X., Jiang F., Jiang Z., Tong S. (2020). Epidemiology of COVID-19 Among Children in China. Pediatrics.

[24] Zhang C., Gu J., Chen Q., Deng N., Li J., Huang L., Zhou X. (2020). Clinical and epidemiological characteristics of pediatric SARS-CoV-2 infections in China: A multicenter case series. PLoS Med..

[25] Ma X., Liu S., Chen L., Zhuang L., Zhang J., Xin Y. (2020). The clinical characteristics of pediatric inpatients with SARS-CoV-2 infection: A meta-analysis and systematic review. J. Med. Virol..

[26] Ding Y., Yan H., Guo W. (2020). Clinical Characteristics of Children With COVID-19: A Meta-Analysis. Front. Pediatr..

[27] Du W., Yu J., Wang H., Zhang X., Zhang S., Li Q., Zhang Z. (2020). Clinical characteristics of COVID-19 in children compared with adults in Shandong Province, China. Infection.

[28] Qiu H., Wu J., Hong L., Luo Y., Song Q., Chen D. (2020). Clinical and epidemiological features of 36 children with coronavirus disease 2019 (COVID-19) in Zhejiang, China: An observational cohort study. Lancet Infect. Dis..

[29] Zhu N., Zhang D., Wang W., Li X., Yang B., Song J., Zhao X., Huang B., Shi W., Lu R. (2020). A Novel Coronavirus from Patients with Pneumonia in China, 2019. N. Engl. J. Med..

[30] Shen K.-L., Yang Y., Wang T., Zhao D., Jiang Y., Jin R., Zheng Y., Xu B., Xie Z., Lin L. (2020). Diagnosis, treatment, and prevention of 2019 novel coronavirus infection in children: Experts’ consensus statement. World J. Pediatr..

[31] Luo S., Zhang X., Xu H. (2020). Don’t Overlook Digestive Symptoms in Patients With 2019 Novel Coronavirus Disease (COVID-19). Clin. Gastroenterol. Hepatol..

[32] Huang C., Wang Y., Li X., Ren L., Zhao J., Hu Y., Zhang L., Fan G., Xu J., Gu X. (2020). Clinical features of patient infected with 2019 novel coronavirus in Wuhan, China. Lancet.

[33] Wang D., Hu B., Hu C., Zhu F., Liu X., Zhang J., Wang B., Xiang H., Cheng Z., Xiong Y. (2020). Clinical Characteristics of 138 Hospitalized Patients With 2019 Novel Coronavirus–Infected Pneumonia in Wuhan, China. JAMA.

[34] Dong Y., Mo X., Hu Y., Qi X., Jiang F., Jiang Z., Tong S. (2020). Epidemiological characteristics of 2143 pediatric patients with 2019 coronavirus disease in China. Pediatrics.

[35] Shekerdemian L.S., Mahmood N.R., Wolfe K.K., Riggs B.J., Ross C.E., McKiernan C.A., Heidemann S.M., Kleinman L.C., Sen A.I., Hall M.W. (2020). Characteristics and Outcomes of Children With Coronavirus Disease 2019 (COVID-19) Infection Admitted to US and Canadian Pediatric Intensive Care Units. JAMA Pediatr..

[36] Zheng F., Liao C., Fan Q.-H., Chen H.-B., Zhao X.-G., Xie Z.-G., Li X.-L., Chen C.-B., Lu X.-X., Liu Z.-S. (2020). Clinical Characteristics of Children with Coronavirus Disease 2019 in Hubei, China. Curr. Med. Sci..

[37] (2020). Decreto del Presidente del Consiglio dei Ministri. https://www.gazzettaufficiale.it/eli/id/2020/03/09/20A01558/sg.

[38] Remuzzi A., Remuzzi G. (2020). COVID-19 and Italy: What next?. Lancet.

[39] Presidenza del Consiglio dei Ministri, Linee di Indirizzo per la Promozione e il Miglioramento della Qualità, della Sicurezza e Dell’appropriatezza degli Interventi Assistenziali in Area Pediatrico-Adolescenziale. http://www.salute.gov.it/portale/news/p3_2_1_1_1.jsp?lingua=italiano&menu=notizie&p=dalministero&id=3256.

[40] Linee di indirizzo nazionali sul triage intraospedaliero (2019). Ministero della salute. http://www.salute.gov.it/imgs/C_17_notizie_3849_listaFile_itemName_1_file.pdf.

[41] Li H., Yu G., Duan H., Fu J., Shu Q. (2020). Changes in Children’s Healthcare Visits During Coronavirus Disease-2019 Pandemic in Hangzhou, China. J. Pediatr..

[42] Baum A., Schwartz M.D. (2020). Admissions to Veterans Affairs Hospitals for Emergency Conditions During the COVID-19 Pandemic. JAMA.

[43] NHS England (2020). A&E Attendances and Emergency Admissions 2019–20: Adjusted Monthly. A&E Time Series. https://www.england.nhs.uk/statistics/statistical-work-areas/ae-waiting-times-and-activity/ae-attendances-and-emergency-admissions-2019-20/.

[44] Raza M.W., Iqbal M.Z., Ahmed M.I., Nawaz T. (2020). Emergency Surgery during Lockdown: Experience at a tertiary care hospital. J. Rawalpindi Med. Coll..

[45] Grandi G., Del Savio M.C., Caroli M., Capobianco G., Dessole F., Tupponi G., Petrillo M., Succu C., Paoletti A.M., Facchinetti F. (2020). The impact of COVID-19 lockdown on admission to gynecological emergency departments: Results from a multicenter Italian study. Int. J. Gynecol. Obstet..

[46] Oseran A.S., Nash D., Kim C., Moisuk S., Lai P.Y., Pyhtila J., Sequist T.D., Wsafy J.H. (2020). Changes in Hospital Admissions for Urgent Conditions During COVID-19 Pandemic. Am. J. Manag. Care.

[47] Rausa E., Kelly M.E., Manfredi R., Riva I., Lucianetti A. (2020). Impact of COVID-19 on attendances to a major emergency department: An Italian perspective. Intern. Med. J..

[48] Gallo O., Locatello L.G., Orlando P., Martelli F., Bruno C., Cilona M., Fancello G., Mani R., Vitali D., Bianco G. (2020). The clinical consequences of the COVID -19 lockdown: A report from an Italian referral ENT department. Laryngoscope.

[49] Ojetti V., Covino M., Brigida M., Petruzziello C., Saviano A., Migneco A., Candelli M., Franceschi F. (2020). Non-COVID Diseases during the Pandemic: Where Have All Other Emergencies Gone?. Medicina.

[50] Bhambhvani H.P., Rodrigues A.J., Yu J.S., Carr J.B., Gephart M.H. (2020). Hospital Volumes of 5 Medical Emergencies in the COVID-19 Pandemic in 2 US Medical Centers. JAMA Intern. Med..

[51] Gissey L.C., Casella G., Russo M.F., Del Corpo G., Iodice A., Lattina I., Ferrari P., Iannone I., Mingoli A., La Torre F. (2020). Impact of COVID-19 outbreak on emergency surgery and emergency department admissions: An Italian level 2 emergency department experience. BJS.

[52] Cheng V.C.-C., Wong S.-C., Chuang V.W.-M., So S.Y.-C., Chen J.H.-K., Sridhar S., To K.K.-W., Chan J.F.-W., Hung I.F.-N., Ho P.-L. (2020). The role of community-wide wearing of face mask for control of coronavirus disease 2019 (COVID-19) epidemic due to SARS-CoV-2. J. Infect..

[53] Cheng V.C.-C., Wong S.-C., Chan V.W.-M., So S.Y.-C., Chen J.H.-K., Yip C.C.-Y., Chan K.-H., Chu H., Chung T.W.-H., Sridhar S. (2020). Air and environmental sampling for SARS-CoV-2 around hospitalized patients with coronavirus disease 2019 (COVID-19). Infect. Control Hosp. Epidemiol..

[54] Leung N.H.L., Chu D.K.W., Shiu E.Y.C., Chan K.-H., McDevitt J.J., Hau B.J.P., Yen H.-L., Li Y., Ip D.K.M., Peiris J.S.M. (2020). Respiratory virus shedding in exhaled breath and efficacy of face masks. Nat. Med..

[55] Kampf G., Brüggemann Y., Kaba H., Steinmann J., Pfaender S., Scheithauer S. (2020). Potential sources, modes of transmission and effectiveness of prevention measures against SARS-CoV-2. J. Hosp. Infect..

[56] Chin A.W.H., Chu J.T.S., Perera M.R., Hui K.P.Y., Yen H.-L., Chan M.C.W., Peiris M., Poon L.L. (2020). Stability of SARS-CoV-2 in different environmental conditions. Lancet Microbe.

[57] Kratzel A., Todt D., V’Kovski P., Steiner S., Gultom M., Thao T.T.N., Ebert N., Holwerda M., Steinmann J., Niemeyer D. (2020). Inactivation of Severe Acute Respiratory Syndrome Coronavirus 2 by WHO-Recommended Hand Rub Formulations and Alcohols. Emerg. Infect. Dis..

[58] Rabenau H., Kampf G., Cinatl J., Doerr H. (2005). Efficacy of various disinfectants against SARS coronavirus. J. Hosp. Infect..

[59] Bae S., Kim M.-C., Kim J.Y., Cha H.-H., Lim J.S., Jung J., Oh D.K., Lee M.-K., Choi S.-H., Sung M. (2020). Effectiveness of Surgical and Cotton Masks in Blocking SARS–CoV-2: A Controlled Comparison in 4 Patients. Ann. Intern. Med..

[60] Anderson R.M., Heesterbeek H., Klinkenberg D., Hollingsworth T.D. (2020). How will country-based mitigation measures influence the course of the COVID-19 epidemic?. Lancet.

[61] Koo J.R., Cook A.R., Park M., Sun Y., Sun H., Lim J.T., Tam C., Dickens B.L. (2020). Interventions to mitigate early spread of SARS-CoV-2 in Singapore: A modelling study. Lancet Infect. Dis..

[62] WHO (2020). Rational use of personal protective equipment for coronavirus disease (COVID-19) and considerations during severe shortages. Interim Guidance.

[63] Rodríguez-Barranco M., Rivas-García L., Quiles J.L., Redondo-Sánchez D., Aranda-Ramírez P., Llopis-González J., Pérez M.J.S., Sanchez-Gonzalez C. (2021). The spread of SARS-CoV-2 in Spain: Hygiene habits, sociodemographic profile, mobility patterns and comorbidities. Environ. Res..

[64] Grüber C., Keil T., Kulig M., Roll S., Wahn U., Wahn V. (2008). The MAS-90 Study Group History of respiratory infections in the first 12 yr among children from a birth cohort. Pediatr. Allergy Immunol..

[65] Blecker S., Jones S.A., Petrilli C.M., Admon A.J., Weerahandi H., Francois F., Horwitz L.I. (2020). Hospitalizations for Chronic Disease and Acute Conditions in the Time of COVID-19. JAMA Intern. Med..

[66] Papadopoulos N.G., Custovic A., Deschildre A., Mathioudakis A.G., Phipatanakul W., Wong G., Xepapadaki P., Agache I., Bacharier L., Bonini M. (2020). Impact of COVID-19 on Pediatric Asthma: Practice Adjustments and Disease Burden. J. Allergy Clin. Immunol. Pr..

[67] Matsumoto K., Saito H. (2020). Does asthma affect morbidity or severity of COVID-19?. J. Allergy Clin. Immunol..

[68] Kimura H., Francisco D., Conway M., Martinez F.D., Vercelli D., Polverino F., Billheimer D., Kraft M. (2020). Type 2 inflammation modulates ACE2 and TMPRSS2 in airway epithelial cells. J. Allergy Clin. Immunol..

[69] Barroso B., Valverde-Monge M., Cañas J.A., Rodrigo-Muñoz J.M., Gonzalez-Cano B., Villalobos-Violan V., Betancor D., Gomez-Cardeñosa A., Vallejo-Chamorro G., Baptista L. (2020). Presenting prevalence, characteristics and outcome of asthmatic patients with T2 diseases in hospitalized subjects with COVID-19 in Madrid, Spain. J. Investig. Allergol. Clin. Immunol..

[70] Domínguez-Ortega J., López-Carrasco V., Barranco P., Ifim M., Luna J.A., Romero D., Quirce S. (2020). Early experiences of SARS-CoV-2 infection in severe asthmatics receiving biologic therapy. J. Allergy Clin. Immunol. Pr..

[71] Chhiba K.D., Patel G.B., Vu T.H.T., Chen M.M., Guo A., Kudlaty E., Mai Q., Yeh C., Muhammad L.N., Harris K.E. (2020). Prevalence and characterization of asthma in hospitalized and nonhospitalized patients with COVID-19. J. Allergy Clin. Immunol..

[72] Rial M.J., Valverde M., Del Pozo V., González-Barcala F.J., Martínez-Rivera C., Muñoz X., Olaguibel J.M., Plaza V., Curto E., Quirce S. (2020). Clinical characteristics in 545 patients with severe asthma on biological treatment during the COVID-19 outbreak. J. Allergy Clin. Immunol. Pr..

[73] Grandbastien M., Piotin A., Godet J., Abessolo-Amougou I., Ederlé C., Enache I., Fraisse P., Hoang T.C.T., Kassegne L., Labani A. (2020). SARS-CoV-2 Pneumonia in Hospitalized Asthmatic Patients Did Not Induce Severe Exacerbation. J. Allergy Clin. Immunol. Pr..

[74] Cosgriff R., Ahern S., Bell S.C., Brownlee K., Burgel P.-R., Byrnes C., Corvol H., Cheng S.Y., Elbert A., Faro A. (2020). A multinational report to characterise SARS-CoV-2 infection in people with cystic fibrosis. J. Cyst. Fibros..

[75] Viganò M., Mantovani L., Cozzolino P., Harari S. (2020). Correction to: Treat all COVID 19‑positive patients, but do not forget those negative with chronic diseases. Intern. Emerg. Med..

[76] Rajkumar R.P. (2020). COVID-19 and mental health: A review of the existing literature. Asian J. Psychiatry.

[77] Panuccio A., Pinto L., Urbino A., Lubrano R., Guidi B., Zampogna S., Cantoni B. I nuovi modelli di Triage. Proceedings of the XII National Congress, Società Italiana di Medicina di Emergenza ed Urgenza Pediatrica (SIMEUP).

[78] Lazzerini M., Barbi E., Apicella A., Marchetti F., Cardinale F., Trobia G. (2020). Delayed access or provision of care in Italy resulting from fear of COVID-19. Lancet Child Adolesc. Health.

[79] Lynn R.M., Avis J.L., Lenton S., Amin-Chowdhury Z., Ladhani S.N. (2020). Delayed access to care and late presentations in children during the COVID-19 pandemic: A snapshot survey of 4075 paediatricians in the UK and Ireland. Arch. Dis. Child..

[80] Jeffery M.M., D’Onofrio G., Paek H., Platts-Mills T.F., Soares W.E., Hoppe J.A., Genes N., Nath B., Melnick E.R. (2020). Trends in Emergency Department Visits and Hospital Admissions in Health Care Systems in 5 States in the First Months of the COVID-19 Pandemic in the US. JAMA Intern. Med..

[81] Thornton J. (2020). Covid-19: A&E visits in England fall by 25% in week after lockdown. BMJ.

[82] Isba R., Edge R., Jenner R., Broughton E., Francis N., Butler J. (2020). Where have all the children gone? Decreases in paediatric emergency department attendances at the start of the COVID-19 pandemic of 2020. Arch. Dis. Child..

